# Evidence-based ergonomic parameters for safer surgical practice: a systematic review

**DOI:** 10.3389/fpubh.2026.1872145

**Published:** 2026-08-13

**Authors:** Jacqueline Wright, Erika Oki, Gonzalo Mariscal, Carlos Barrios

**Affiliations:** 1 Mizuho OSI, Union City, CA, United States; 2 Institute for Research on Musculoskeletal Disorders, Catholic University of Valencia, Valencia, Spain

**Keywords:** ergonomic interventions, musculoskeletal disorders, operating room design, postural biomechanics, surgeon ergonomics

## Abstract

**Introduction:**

Work-related musculoskeletal disorders are highly prevalent among surgeons and are primarily associated with sustained non-neutral postures, inadequate visualization, improper table height, and insufficient adaptation to individual anthropometry; therefore, this systematic review aimed to synthesize evidence on ergonomic risk factors and define evidence-based parameters for safer surgical practice.

**Methods:**

A comprehensive systematic search of multiple databases was conducted using predefined eligibility criteria, including studies evaluating surgeon anthropometry, visualization techniques, table height, muscle activity, posture, and ergonomic accessories; findings were qualitatively synthesized across predetermined ergonomic domains such as neutral posture, electromyographic fatigue, spinal kinematics, and ergonomic modifications.

**Results:**

Twenty-six studies across laparoscopic, endoscopic, microscopic, otolaryngologic, neurosurgical, and urological procedures were included. Beneficial ergonomic ranges included cervical flexion of 0–10°, shoulder abduction below 30°, trunk inclination under 20°, and minimal neck rotation. Ergonomic redesign reduced trapezius fatigue from 100 to 42%, while monitor-based endoscopy decreased trapezius activity to approximately one-third compared with direct eyepiece viewing. Optimal table height varied widely, commonly defined relative to elbow height or surgeon stature, with required ranges (29–122 cm) exceeding standard table capabilities.

**Discussion:**

Surgeon ergonomics is strongly influenced by operating room design, visualization setup, and anthropometric variability, and current equipment frequently fails to meet evidence-based requirements, supporting the need for redesigned surgical tables, improved visualization systems, and integrated ergonomic accessories to reduce strain and enhance long-term occupational sustainability.

## 1 Introduction

Surgeons usually work in an environment characterized by prolonged static postures, repetitive fine motor activity, sustained visual concentration, and frequent non-neutral positioning of the neck, shoulders, trunk, and upper limbs (1–3). These exposures occur across open, endoscopic, microscopic, and minimally invasive procedures, although minimally invasive surgery may impose additional ergonomic constraints through fixed monitor locations, long instruments, restricted degrees of freedom, and visually mediated hand movements rather than direct line-of-sight operating (4, 5). As a result, surgeon ergonomics has emerged as an important occupational health issue with implications not only for physical well-being, but also for performance, endurance, and career longevity.

The burden of work-related musculoskeletal disorders among surgeons is substantial. In surgeons performing minimally invasive procedures, reported physical complaints have reached 73–88% in some series (4). More broadly, a systematic review and meta-analysis of surgeons and interventionalists found pooled pain prevalence estimates ranging from 35 to 60% and reported that 12% of affected physicians required leave of absence, practice restriction or modification, or early retirement because of work-related musculoskeletal disorders (6). Specialty-specific evidence points in the same direction; for example, a recent systematic review in orthopaedic surgery reported career prevalence estimates ranging from 37 to 97% (7).

Despite growing awareness of this problem, structured ergonomic prevention remains limited. Survey data from U.S. Surgical training programs revealed that merely 1.5% provided formal education in surgical ergonomics, while 25.4% offered informal instruction, suggesting that numerous surgeons start practice without comprehensive training in posture optimization, table height adjustment, monitor positioning, or equipment arrangement (8). Existing reviews have helped define the prevalence of surgeon musculoskeletal injury and have evaluated interventions such as ergonomics training and intraoperative microbreaks (9, 10), while more recent reviews have summarized ergonomic concerns across open, laparoscopic, and robotic surgery (1). Nevertheless, the literature remains limited in a practically oriented synthesis that converts objective ergonomic evidence into specific operational parameters, including neutral postural zones, optimal table heights, visualization strategies, and accessory modifications.

Accordingly, this systematic review sought to consolidate the existing knowledge on surgeon ergonomics in various operating environments, focusing specifically on postural balance, muscular fatigue, surgical table height, visualization techniques, ergonomic accessories, and anthropometric diversity. This review aims to identify evidence-based ergonomic principles that can guide operating room design, equipment development, and daily surgical practice by synthesizing findings from observational, experimental, and simulation-based investigations.

## 2 Methods

### 2.1 Protocol registration

Our systematic review protocol was registered in OSF [10.17605/OSF.IO/CM2J9]. The review was conducted in accordance with the Preferred Reporting Items for Systematic Reviews and Meta-Analyses (PRISMA) statement and the methodological guidance of the Cochrane Handbook for Systematic Reviews (11, 12).

### 2.2 Data sources and search strategy

A comprehensive search of PubMed (MEDLINE), Scopus, and Web of Science was conducted from database initiation to February 2026, without applying study-design filters at the search stage. The search technique integrated controlled vocabulary and free-text terms pertaining to surgeons, ergonomics, posture, musculoskeletal strain, tiredness, operating table height, monitor positioning, visualization systems, and ergonomic accessories. Boolean operators (AND/OR) were used to combine Medical Subject Headings (MeSH) and free-text terms within and across these concept blocks (e.g., surgeon* AND ergonomic* AND [posture OR “table height” OR “monitor position”]); the complete, database-specific search strings, the exact date of the final search (28 February 2026), and confirmation that no grey literature sources (conference abstracts, theses, or preprint servers) were searched are all reported in Supplementary Table 1. Reference lists of relevant reviews and included studies were also screened to identify additional eligible records. The full search strategy for each database is provided in Supplementary Table 1.

### 2.3 Eligibility criteria

Those original studies that evaluate ergonomic factors affecting surgeons or surgical trainees during operative or simulated surgical tasks were included. Eligible studies enrolled participants involved in any surgical specialty and assessed at least one ergonomics-related exposure, intervention, or configuration, such as table height, monitor position, body posture, visualization modality, instrument design, ergonomic supports, or operating-room setup. Studies should report at least one relevant ergonomic outcome, including but not limited to postural angles, neutral-zone measurements, electromyographic activity, fatigue, pain, discomfort, spinal kinematics, workload, or posture-assessment scores.

broad range of study designs were considered, including randomized crossover trials, prospective or retrospective observational studies, experimental simulation studies, comparative studies, and participatory design studies, provided they reported original human data. Questionnaire- and survey-based studies were also eligible, provided they reported a surgeon-specific ergonomic exposure or outcome as defined above.

Non-human, cadaveric, or purely technical bench studies without human ergonomic assessment were excluded. Similarly, narrative reviews, systematic reviews, meta-analyses, editorials, letters, conference abstracts, guidelines, and book chapters were also excluded. Studies with overlapping datasets, in which case the most complete or most recent report was retained, and studies not published in English were ignored. Finally, studies not reporting surgeon-specific ergonomic outcomes were also excluded.

### 2.4 Study selection

All retrieved records were imported into Rayyan for screening, and duplicates were removed before eligibility assessment. Two reviewers independently screened titles and abstracts to identify potentially relevant studies. Full texts of potentially eligible articles were then reviewed in detail against the predefined inclusion and exclusion criteria. Disagreements at any stage were resolved through discussion, and when necessary, by consultation with a third senior reviewer.

### 2.5 Data extraction

Data extraction was performed independently by two reviewers using a piloted standardized Excel sheet developed specifically for this review. For each included study, we collected the following information: country, study design, surgical specialty or procedure type, setting (operative or simulated), sample size, participant type, ergonomic assessment method, intervention or exposure of interest, reported outcomes, and principal findings.

Where available, details were extracted if concern to surgeon anthropometry, such as height or body size; operating configuration variables, including table height, monitor height or position, visualization method, and use of ergonomic accessories; and outcome measures such as RULA or REBA scores, EMG activity, posture angles, fatigue indices, pain scales, and performance-related metrics.

### 2.6 Quality assessment

Methodological quality of the included studies was assessed independently by two reviewers using the NIH Quality Assessment Tool for Observational Cohort and Cross-Sectional Studies (13). This tool was selected because most included studies were observational, simulation-based, or non-randomized in design. The assessment considered key domains including clarity of the research question, definition of the study population, participation rate, sample selection, exposure and outcome measurement, timeframe, blinding of outcome assessors, loss to follow-up where applicable, and control of confounding variables.

### 2.7 Data synthesis

Because of substantial clinical, methodological, and statistical heterogeneity across the included studies, a quantitative meta-analysis was not considered appropriate. Heterogeneity arose from differences in surgical specialties, ergonomic exposures and interventions, study designs, task settings, assessment tools, and reported outcome measures. Therefore, findings were synthesized narratively.

The narrative synthesis was structured according to prespecified thematic domains. These included optimal postural angles and neutral working zones, muscular fatigue and electromyographic findings, optimal surgical table heights, the effects of visualization methods on spinal kinematics, the role of ergonomic accessories and procedural modifications, and the influence of surgeon anthropometry and individual variability. Where possible, findings were summarized using descriptive statistics and tabulated to highlight recurrent ergonomic patterns, proposed threshold values, and the magnitude of benefit associated with ergonomic interventions. Focus was placed on identifying consistent evidence across studies and translating these findings into practical recommendations for surgical-table design and operating-room configuration.

## 3 Results

### 3.1 Study selection and baseline characteristics

**Figure 1:**
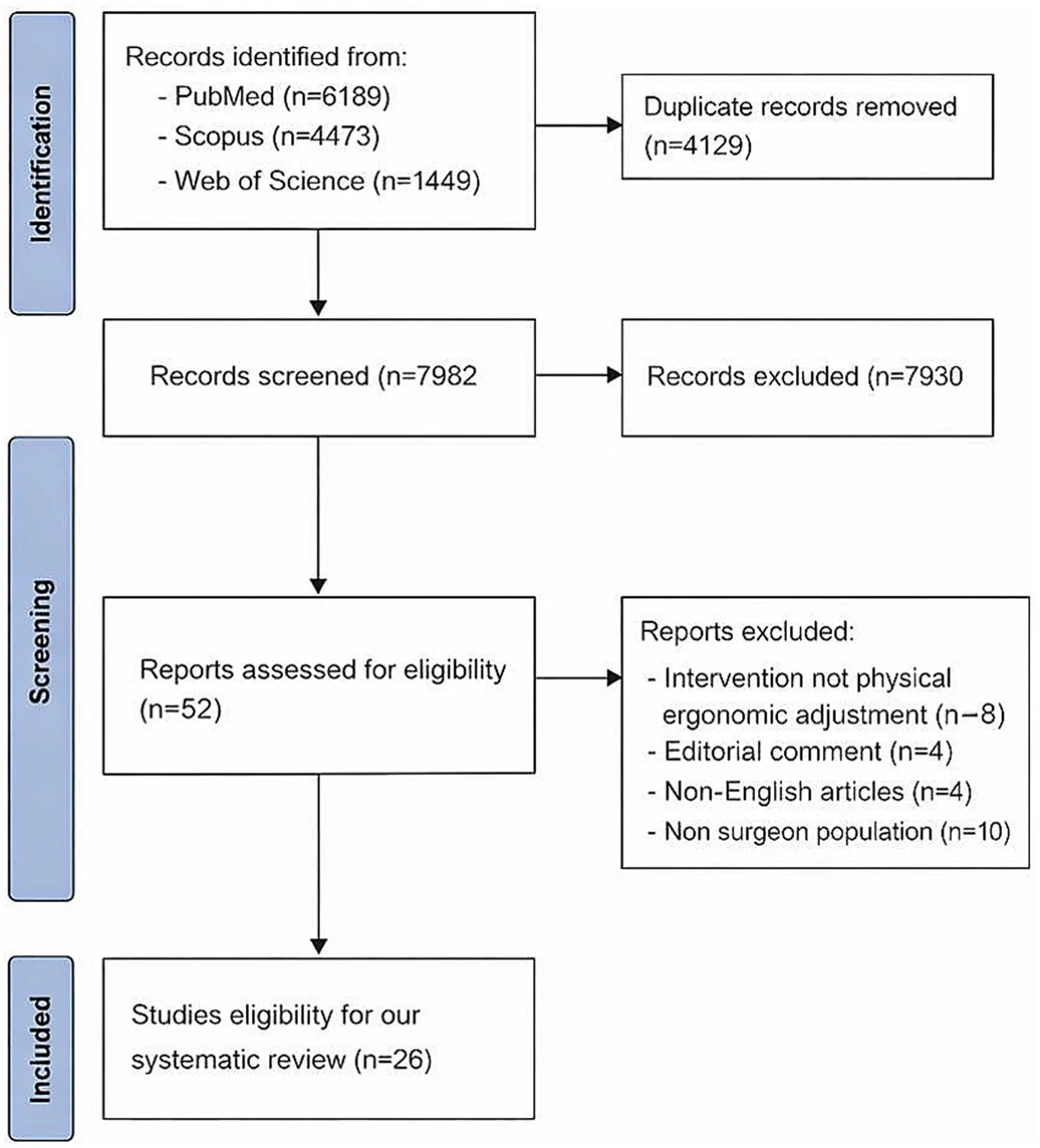
PRISMA flow diagram of study selection.

Database searching identified 12,111 potentially relevant records across all searched databases. After removal of 4,129 duplicates, 7,982 unique records were screened at title and abstract level. Of these, 7,930 records were excluded at the screening stage, leaving 52 reports assessed for full-text eligibility. Following detailed eligibility assessment against predefined inclusion and exclusion criteria, 26 studies met all inclusion criteria and were included in the final qualitative synthesis (14–39) (Figure 1).

The 26 included studies represented diverse surgical specialties and methodological approaches, providing comprehensive coverage of surgical ergonomics literature. Laparoscopic surgery was the primary focus of the analysis (n = 10 studies), highlighting the widespread adoption of minimally invasive techniques and their associated ergonomic challenges. Five studies examined endoscopic procedures, four examined microsurgery and neurosurgery, three examined otolaryngology treatments, and four examined urological procedures. The study designs comprised six randomized crossover trials, 12 prospective observational studies, 5 experimental simulation studies, and 3 participatory design studies. Sample sizes varied from 2 to 128 participants per trial, predominantly including between 8 and 30 surgeons.

Table 1 presents the baseline characteristics of all 26 included research, offering a detailed overview of study populations, methodology, and principal conclusions. Assessment methods varied substantially across studies and included state-of-the-art three-dimensional motion capture systems (Vicon, IMU sensors), surface electromyographic monitoring of multiple muscle groups, validated ergonomic assessment tools (Rapid Upper Limb Assessment [RULA], Rapid Entire Body Assessment [REBA], three-dimensional Static Strength Prediction Program [3D-SSPP]), video-based postural analysis using standardized coding systems, and validated subjective pain and fatigue scales.

**Table 1:** Baseline characteristics of the 26 included studies.

Author, Year	Design	Surgery	n	Methods	Outcome	Key Finding
Baird et al., 2021 (14)	Prospective	Laryngeal (ALS)	8	RULA, EMG, Pain	Posture score	Best RULA = 4.5 frontal/close/neutral
Kranenbur and Gossot, 2004 (18)	Observational	VATS	15 ops	Observation	Neutral zones	Table 0.7–0.8x elbow height
Luttmann et al., 1996 (26)	EMG field study	Urology TUR	14 ops	EMG-JASA	Fatigue	78.6% trapezius fatigue
Luttmann et al., 1996 (27)	Comparative	Urology TUR	15 ops	EMG	EMG reduction	Monitor reduces trapezius to 1/3
Luttmann et al., 1998 (24)	Prospective 3-part	Urology TUR	41 ops	EMG	Fatigue change	100- > 42% fatigue after redesign
Matern et al., 2005 (23)	Randomized exp	Lap simulation	18	Cervical EMG	EMG activity	Frontal monitor = lowest strain
Shimizu et al., 2020 (38)	Observational	Neuro/Spine	8	Observation	Fatigue	75% reduced fatigue with adjustment
Statham et al., 2010 (31)	Prospective case-ctrl	Microlaryngoscopy	3	RULA, 3D-SSPP	Spinal load	Arm support reduces L5/S1 4x
Van Det et al., 2008 (25)	Crossover trial	Lap cholecystectomy	48	Video, VAS	Neck rotation	MIS suite reduces rotation 47%
Vereczkei et al., 2004 (22)	Prospective obs	Lap cholecystectomy	20 ops	Video-PCMAN	Trunk rotation	Lateral monitor reduces trunk 39%
Aitchison et al., 2016 (36)	Prospective obs	Gyn laparoscopy	18/128	Video analysis	Height correlation	Shorter surgeons worse (rs = −0.49)
Luttmann et al., 2009 (20)	Comparative obs	Urology endoscopy	5/19 ops	Video coding	Posture	Monitor allows upright posture
Park et al., 2012 (15)	Randomized cross	Spine simulation	12	Vicon 3D	Spinal angles	Loupe + higher table = natural
Park et al., 2014 (33)	Randomized cross	Spine simulation	18	Vicon 3D	Spinal angles	Microscope closest to natural
Berquer et al., 2001 (17)	Experimental RCT	Lap simulation	21	EMG, VAS	Optimal height	Elbow level or -10 cm; 64–77 cm
Veelen et al., 2002 (19)	Experimental	Lap simulation	8	Joint angles, VAS	Height formula	Factor 0.7–0.8 x elbow height
Kaur, 2008 (32)	Observational exp	Lap simulation	25	Joint movements	Height formula	Formula: height x 0.49
Manasnayakorn et al., 2008 (21)	Experimental 2-part	HALS simulation	10	EMG 14 muscles	Optimal height	Elbow level to +5 cm
Matern et al., 2001 (16)	Simulated model	Laparoscopy	2	Calculations	Required range	Range needed: 29–122 cm
Azimuddin et al., 2017 (14)	Quality assessment	ENT seated	5 tables	Measurements	Table adequacy	64–77 cm recommended
Kelts et al., 2015 (35)	Cross-sectional	ESS	8	Monitor height	System adequacy	Only CMB systems adequate
Abramovic et al., 2021 (29)	Cross-sect workshop	Neurosurg simulation	34	Posture analysis	HMD effect	HMD: 0 deg. median displacement
Albayrak et al., 2007 (34)	Participatory design	Open/Laparoscopic	7	EMG	Support effect	Body support: 40–77% EMG reduction
Fan eta al., (39)	Randomized cross	Simulated surgery	19	IMU, EMG	Loupe comparison	Prismatic: 13–26 deg. head reduction
Hu et al., 2012 (28)	Case study	Lap nephrectomy	2	Observations	Platform effect	Platform: 24% fewer adjustments
Maithel et al., 2004 (30)	Randomized cross	Lap simulation	30	EMG, performance	HMD vs. VMD	HMD better smoothness, VMD less fatigue

Geographic distribution of included studies showed substantial representation from European centers (Germany, Netherlands, United Kingdom, Hungary), North American institutions (United States, Canada), and Asian centers (Japan, India). Publication years spanned from 1996 to 2021, indicating a persistent scholarly focus on surgical ergonomics for almost 20 years.

### 3.2 Risk of bias assessment

Risk of bias assessment using the NIH Quality Assessment Tool for Observational Cohort and Cross-Sectional Studies demonstrated overall moderate methodological quality across the included studies. Out of the evaluated studies, 8 were rated as good quality (scores 11–14), 12 as fair quality (scores 7.5–10.5), and 5 as poor quality (scores ≤7). Good-quality studies—such as those by Fan et al. (39), Maithel et al. (30), Manasnayakorn et al. (21), Matern et al. (23), and several Luttmann studies generally demonstrated clearly defined research questions, well-characterized populations, valid and consistent exposure and outcome measurements, and appropriate consideration of confounding variables. Many studies lacked adequate participation rates or did not report them, did not justify sample size or statistical power, and rarely assessed exposures longitudinally, which reduced internal validity. Additionally, blinding of outcome assessors and repeated exposure assessment were seldom reported, and loss-to-follow-up information was frequently absent in observational designs. Despite these limitations, the predominance of fair-to-good quality studies suggests that the available evidence provides a reasonably reliable but methodologically heterogeneous basis for evaluating the association between the studied exposures and outcomes Supplementary Table 2.

### 3.3 Optimal postural angles and neutral zones

**Figure 2:**
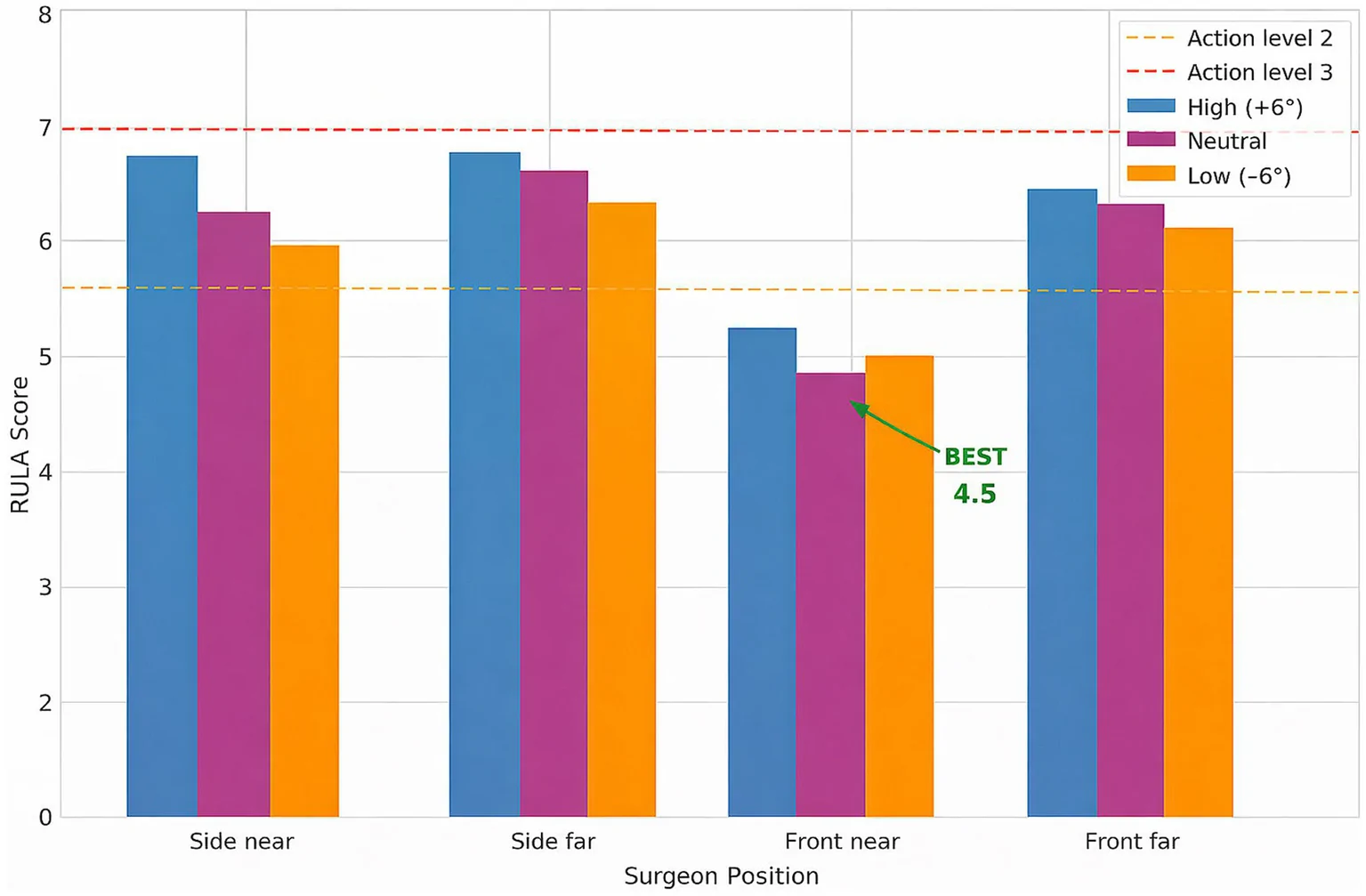
RULA scores by surgeon position and table height configuration.

Analysis of postural data from 15 studies enabled the establishment of evidence-based neutral zones for surgical practice. Figure 2 illustrates that RULA (Rapid Upper Limb Assessment) scores exhibited considerable variation depending on surgery position, table height configuration, and monitor positioning. The RULA methodology has been successfully adapted for surgical contexts and provides a validated framework for evaluating cumulative postural risk. Baird et al. (37) conducted prospective experimental investigation of laryngeal surgery with adjustable laryngoscope suspension (ALS), revealing optimal RULA scores of 4.5 in the frontal-close-neutral position, compared with 6–7 for suboptimal configurations. These findings indicate that modest modifications to surgical configuration can yield significant improvements in postural risk assessment.

The ideal neck flexion angle for cervical spine alignment is 0–10 degrees, which corresponds to the region of minimal muscle effort, when static holding forces are near their physiological minimum. A permissible range of 15–45 degrees was corroborated by two independent studies (18, 37). This acceptable range acknowledges the practical necessity that numerous surgical procedures demand a degree of visual accommodation, requiring cervical flexion beyond the neutral position. Neck flexion above 45 degrees was consistently associated with markedly elevated trapezius electromyographic activity, increased subjective pain ratings, and a rapid onset of tiredness across multiple independent trials. The trapezius muscle, which is crucial for stabilizing neck posture, is particularly sensitive to a forward head posture. Ideally, neck rotation should be kept at 0 degrees relative to the trunk, with any deviations limited to less than 15 degrees. It was also found that lateral monitor placement during.

The shoulder posture demonstrated optimal abduction angles of 0–15 degrees, with an acceptable range up to 30 degrees; beyond this, the cumulative risk of injury markedly increases. It was observed substantial increases in deltoid and trapezius EMG activity during shoulder abduction beyond 30 degrees during endoscopic operations, with further abduction beyond this limit associated with a rapid onset of fatigue (27). The correlation between shoulder abduction and muscular tension is non-linear, with significantly greater elevations in EMG activity at higher abduction angles. Optimal elbow flexion ranged from 80 to 100 degrees, aligning with the mechanical advantage of the elbow flexors and extensors. An acceptable working range of 30–130 degrees has been established showing that positions outside a certain range increased strain on either the flexor or extensor muscles, depending on the direction of the deviation (17, 19). Regarding wrist angles, the best angles for deviation were between 0 and 5 degrees, with acceptable extension up to 25 degrees (34, 38).

Trunk positioning demonstrated ideal inclination angles of 0–10 degrees forward, with an acceptable range extending to 20 degrees. Indicating that trunk flexion above 20 degrees correlated with significantly heightened paraspinal muscle activation and expedited lumbar fatigue (15, 20). The detailed optimal postural angles and neutral zones are presented in Table 2.

**Table 2:** Optimal postural angles and neutral zones.

Body segment	Optimal range	Acceptable	Evidence source
Neck flexion	0–10 deg	15–45 deg	(18, 37)
Neck rotation	0 deg	<15 deg	(22, 23, 25)
Shoulder abduction	0–15 deg	<30 deg	(26, 31)
Elbow flexion	80–100 deg	30–130 deg	(17, 19)
Wrist deviation	0–5 deg	<25 deg	(34, 38)
Trunk inclination	0–10 deg	<20 deg	(15, 20)

**Figure 3:**
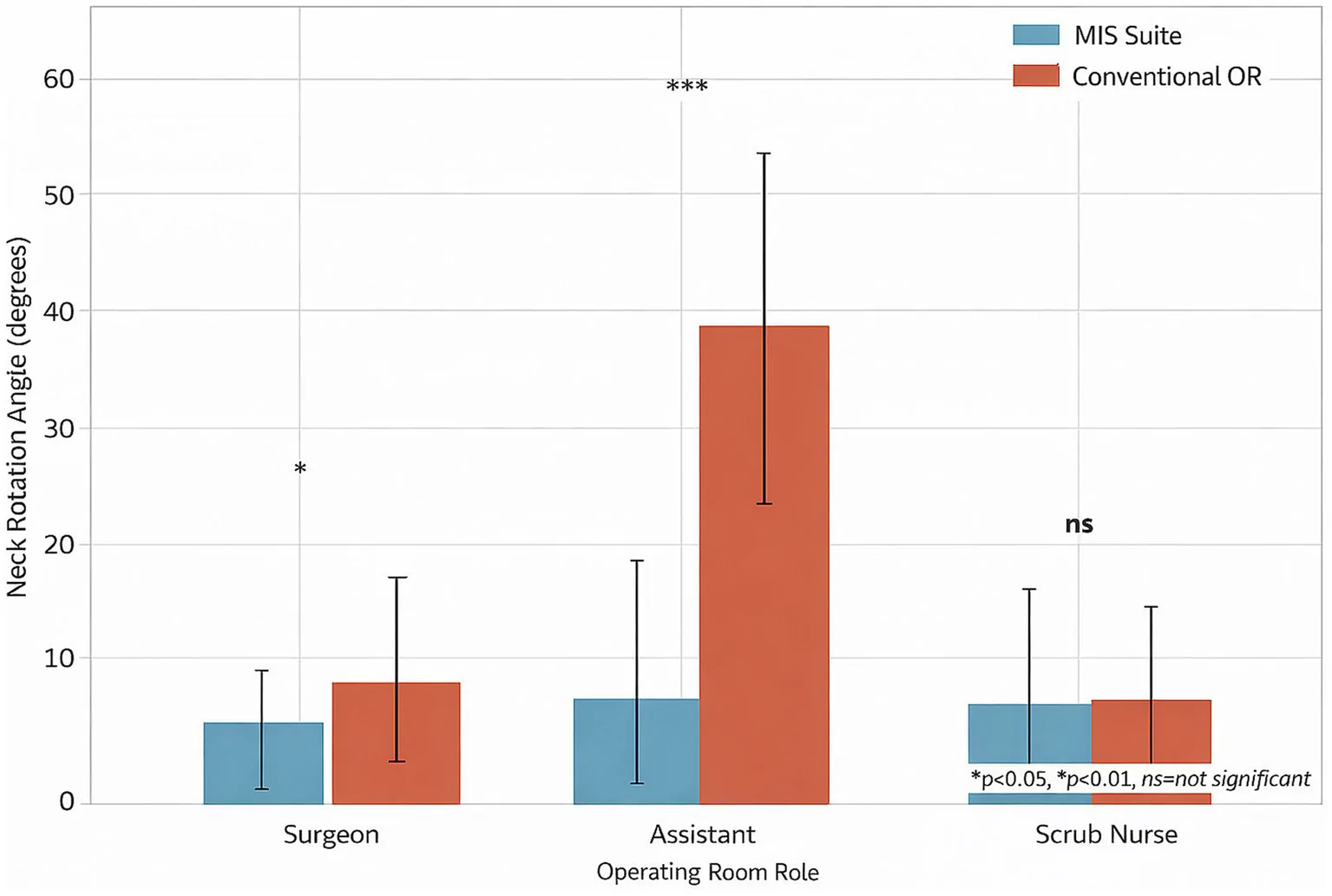
Neck rotation by operating room setup.

One study demonstrated the ergonomic superiority of a dedicated minimally invasive surgery (MIS) suite over conventional operating rooms (OR) in reducing neck rotation angles during spine-relevant procedures (25). While assistants saw significant reductions in the MIS suite (from about 50° to less than 20°, *p* < 0.05, large effect size d = 2.37), surgeons exhibited minor rotations (around 10–20°) in both configurations (Figure 3). Scrub nurses showed non-significant improvements (~30° to 20°), emphasizing setup-specific advantages for extended spine procedures where assistant postures affect MSD risk and team productivity.

### 3.4 Muscular fatigue and EMG findings

**Figure 4:**
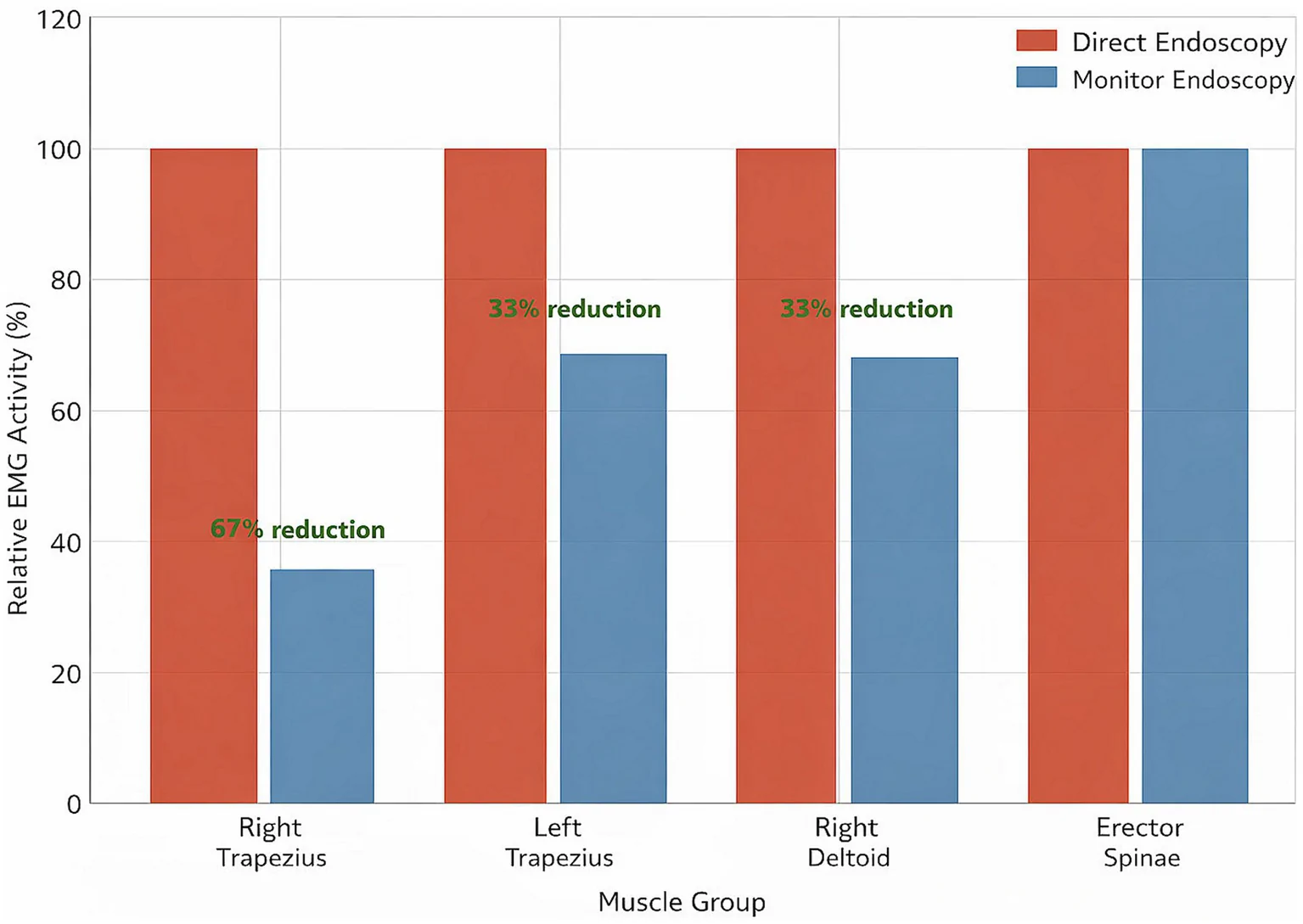
Reduction in trapezius EMG activity with monitor-based endoscopy.

Eight studies used electromyography to assess muscular workload during surgical tasks. In surgical ergonomics research, surface electromyography (sEMG) is particularly valuable because it enables real-time measurement of muscle activation, tracks fatigue over time, and evaluates the effects of ergonomic interventions on specific muscle groups. Figure 4 shows that visualization techniques and ergonomic changes have big and clinically important effects on muscle activity.

Monitor-based endoscopy marked a major advance in surgical ergonomics. It has been found that monitor viewing reduced trapezius muscle activity to nearly one-third of that seen with direct eyepiece visualization (*p* < 0.001) (27). This 67% reduction is one of the largest effect sizes reported in the field and likely reflects the elimination of cervical flexion required for eyepiece viewing, allowing a more neutral head posture.

In addition, a randomized laparoscopic simulation study, showed that frontal monitor placement minimized cervical muscle strain, whereas lateral positioning increased trapezius activity by 40–60%, depending on the angle (23). And there are long-term advantages of ergonomic redesign by a prospective three-component intervention that included optimal monitor positioning, adjustment of work surface height, and modification of instrument handles (24). Post-implementation, the percentage of surgeons experiencing substantial trapezius fatigue decreased from 100 to 42%, with enhancements maintained at the 6-month follow-up, suggesting a lasting ergonomic advantage and a possible decrease in cumulative injury risk (24).

### 3.5 Optimal surgical table heights

**Figure 5:**
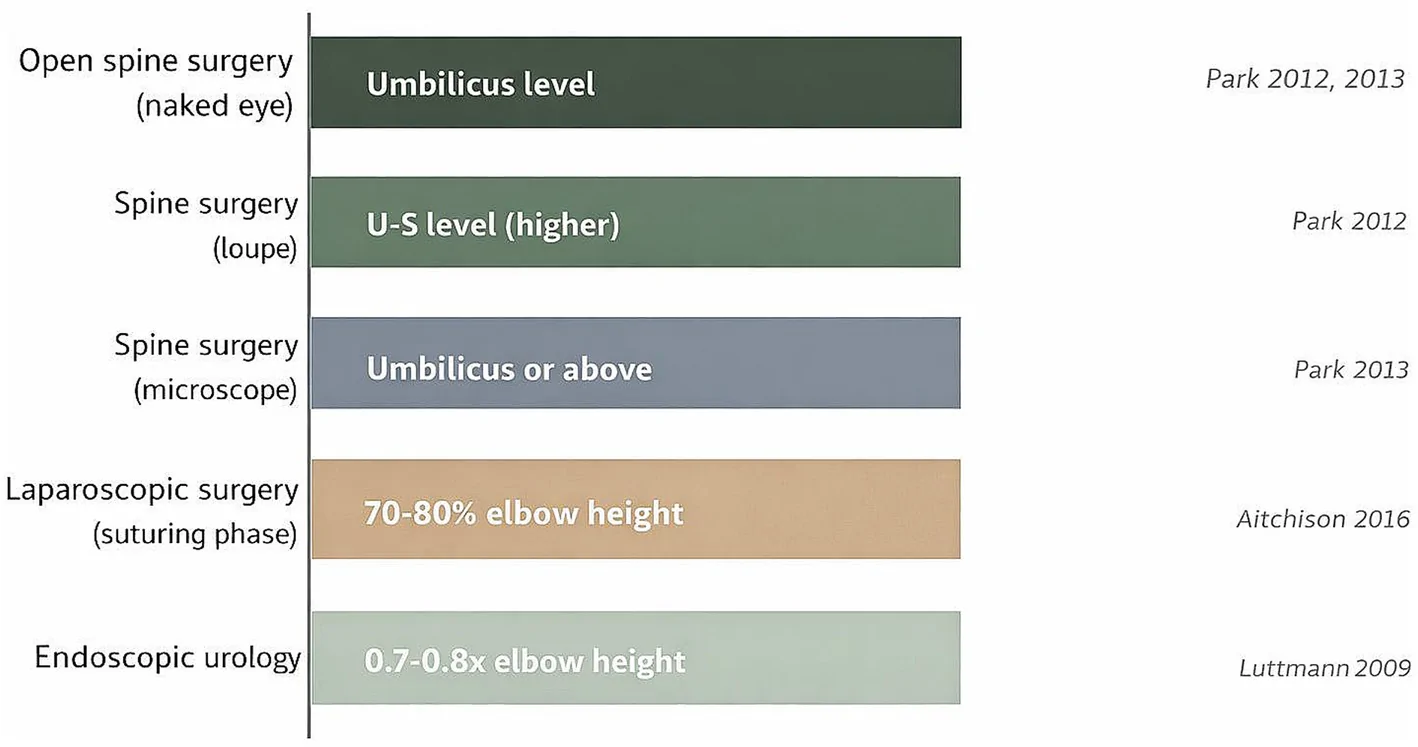
Optimal surgical table height ranges by surgery type.

Seven studies specifically addressed surgical table height optimization, providing formula-based recommendations and absolute height values (Figure 5).

As shown in Table 3, optimal table heights varied by surgical procedure and surgeon anthropometry. For laparoscopic surgery, a factor of 0.7–0.8 times elbow height has been stablished as the optimal table position, typically corresponding to 64–77 cm for average-height surgeons (19). As an alternative, multiplying the height by 0.49, yielded similar optimal heights of 80–86 cm for standing laparoscopic procedures (32). These findings were confirmed in a randomized experimental study that optimal table height approximated elbow level or 10 cm below (17).

**Table 3:** Optimal surgical table heights by surgery type.

Surgery type	Optimal height	Formula	Source
Laparoscopy (standing)	64–77 cm	0.7–0.8 × elbow	(17, 19)
Laparoscopy (general)	80–86 cm	Height × 0.49	(32)
Open surgery	70–90 cm	Elbow height	(17)
HALS	65–75 cm	Hand level to +5 cm	(21)
ENT (seated)	64–77 cm	Chair height	(14, 35)
Spine surgery	90–105 cm	Eye-level access	(15, 33)
Required range	29–122 cm	All anthropometries	(16)

Critically, it was calculated that the required table height range to accommodate all surgeon anthropometries across standing and seated procedures extends from 29 cm to 122 cm (16). This figure derives from a single small simulation study of only two surgeons published in 2001 (16) and should therefore be interpreted with caution rather than as a precise, generalizable target across surgical specialties. This represents a fundamental mismatch with current surgical table capabilities, which typically offer adjustment ranges of only 55–100 cm. This gap of approximately 50% in required versus available range represents a significant limitation of current surgical table designs. For seated ENT procedures the recommended heights of 64–77 cm were appropriate (14).

### 3.6 Impact of visualization methods on spinal kinematics

**Figure 6:**
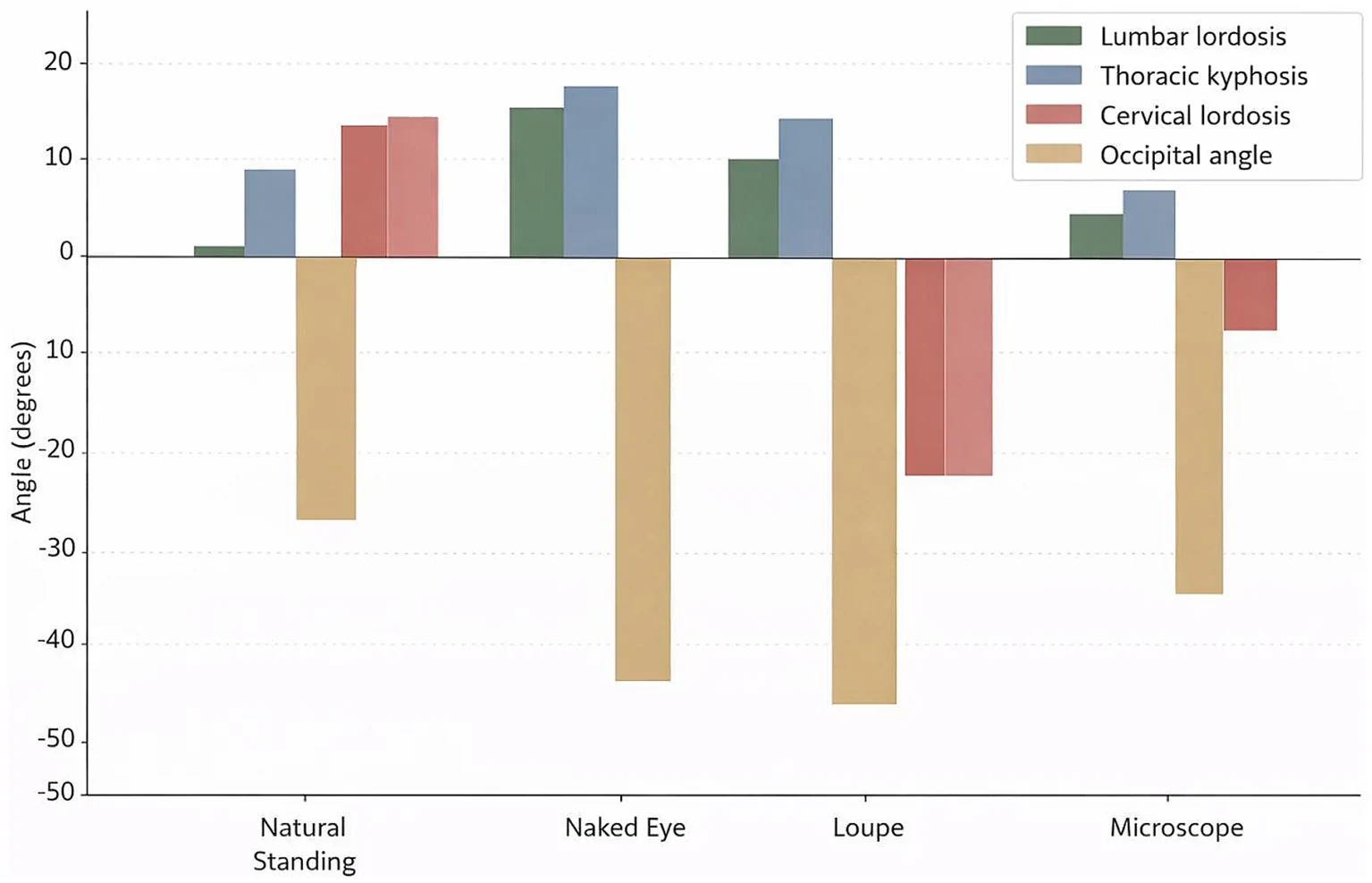
Spinal angles according to visualization method.

Four studies specifically compared spinal kinematics across different visualization methods using three-dimensional motion capture systems (Figure 6).

Natural standing position served as the reference standard, with measured angles of lumbar 1.4–1.9 degrees, thoracic 10.9–11.6 degrees, and cervical −24.6 to −25.7 degrees (negative indicating extension). It has been demonstrated that microscope-assisted surgery maintained spinal angles closest to this natural standing position across all three spinal regions, with statistically significant differences compared to loupe and naked eye visualization (*p* < 0.05) (33).

Loupe magnification produced moderate deviations from natural posture, with lumbar flexion increasing to 16.1 degrees, thoracic flexion to 18.9 degrees, and cervical angle shifting to −6.2 degrees. It was confirmed that loupe use with higher table positioning approximated natural standing more closely than lower table heights (15).

Naked eye visualization required the greatest postural deviation, with lumbar flexion reaching 23.7 degrees, thoracic flexion 26.4 degrees, and cervical angle shifting to positive 2.3 degrees (flexion rather than extension). These deviations were statistically significant across all measured regions (*p* < 0.001).

The impact of integrated MIS suite design on neck rotation during laparoscopic cholecystectomy has been evaluated with a crossover trial that demonstrated that optimized monitor positioning reduced neck rotation by 47% compared to conventional operating room configurations, with corresponding improvements in surgeon comfort scores (25). This was confirmed in another study, demonstrating that lateral monitor placement reduced trunk rotation by 39% compared to midline positioning during laparoscopic cholecystectomy, as assessed by video-based postural coding software (PCMAN) analysis (22).

### 3.7 Ergonomic accessories and modifications

**Figure 7:**
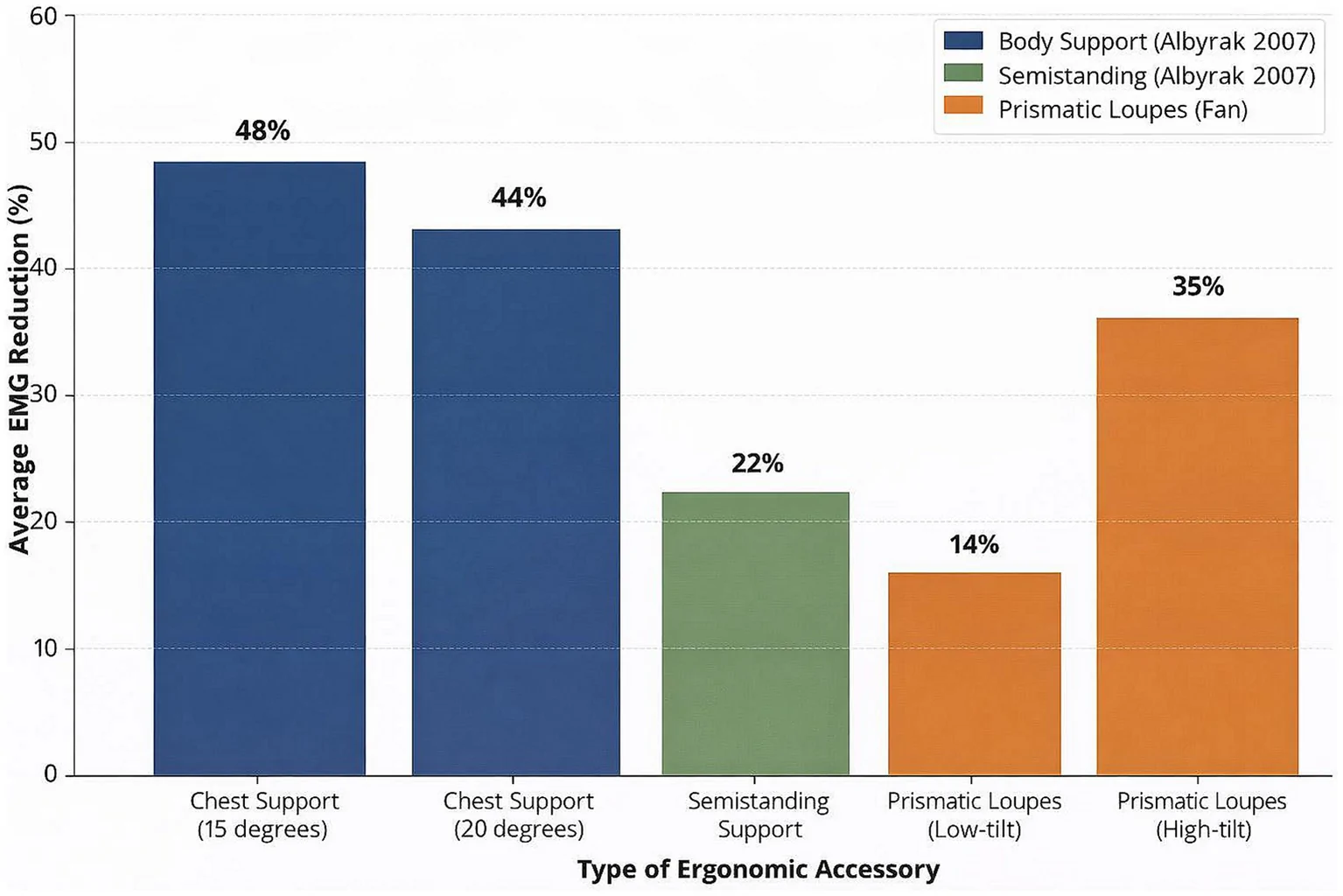
Effect of ergonomic accessories on muscular strain.

Five studies evaluated the impact of ergonomic accessories on musculoskeletal strain. As summarized in Figure 7, these modifications demonstrated substantial benefits across multiple outcome measures.

In a clinical study design evaluating body supports during open and laparoscopic surgery. Using EMG monitoring of 14 different muscles, the study demonstrated reductions of 40–77% in paraspinal and cervical muscle activity with properly positioned body supports. The chest support configuration was particularly effective for procedures requiring sustained forward lean (34).

In addition, in a randomized crossover trial comparing standard and prismatic loupes during simulated surgical tasks. Inertial measurement unit (IMU) data combined with EMG demonstrated that prismatic loupes reduced head flexion by 13–26 degrees compared to standard loupes, with corresponding reductions in cervical muscle activity of up to 42% (39).

In a case study, it was also demonstrated that platforms reduced the need for table height adjustments by 24% and enabled optimal positioning for shorter surgeons who would otherwise be unable to achieve appropriate visual and manual access (28).

While comparing head-mounted displays (HMD) versus video monitor displays (VMD) for laparoscopic surgery in a randomized crossover design (30), HMD demonstrated better task performance smoothness, VMD was associated with lower subjective fatigue ratings. Moreover, HMD has proven to maintained 0 degrees median head displacement compared to significant flexion with conventional visualization (29).

### 3.8 Surgeon anthropometry and individual variation

**Figure 8:**
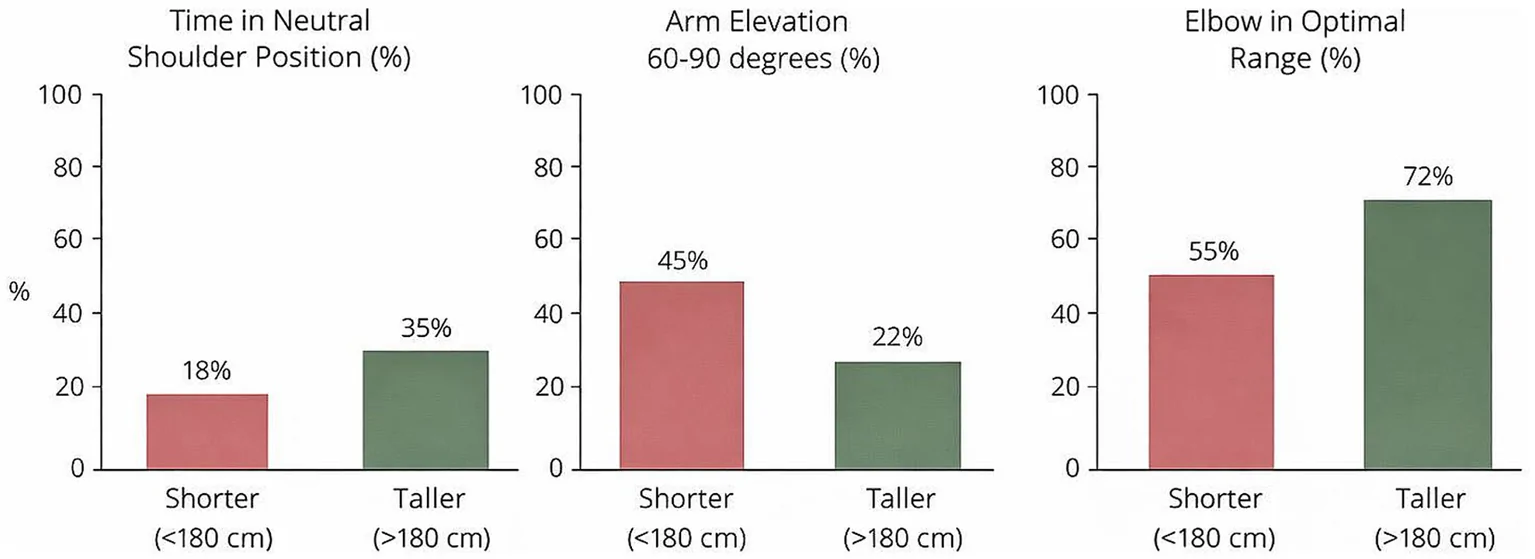
Relationship between surgeon anthropometry and optimal setup.

Four studies specifically evaluated the relationship between surgeon anthropometry and optimal positioning parameters. As illustrated in Figure 8, surgeon height emerged as a significant determinant of optimal table configuration.

In a prospective observational study of 18 surgeons performing 128 gynecological laparoscopic procedures. Video-based postural analysis revealed significant correlations between surgeon height and postural outcomes, with correlation coefficients ranging from rs = −0.488 to rs = −0.553. Shorter surgeons consistently exhibited worse postural angles compared to taller colleagues when using identical table configurations (36).

It has been confirmed that shorter surgeons required proportionally greater postural compensation, and that monitor-based visualization enabled more upright posture across all height groups (20). And that individualized adjustment reduced reported fatigue by 75% compared to standardized configurations (38).

Based on the synthesized evidence, Table 4 presents a comprehensive design recommendations for surgical tables.

**Table 4:** Evidence-based design recommendations for surgical tables.

Parameter	Current standard	Recommended	Evidence level
Height range	55–100 cm	29–122 cm	Strong (16, 23)
Height adjustment	Electric/hydraulic	Electric, fine control	Moderate (17)
Trendelenburg	−30 to +30 deg	Maintain range	Moderate
Support integration	Optional accessories	Built-in mounts	Strong (34)
Monitor mounts	Separate systems	Integrated, adjustable	Strong (23)
Platform compatibility	Limited	Universal design	Moderate (28)

### 3.9 Table height requirements in spine surgery

**Figure 9:**
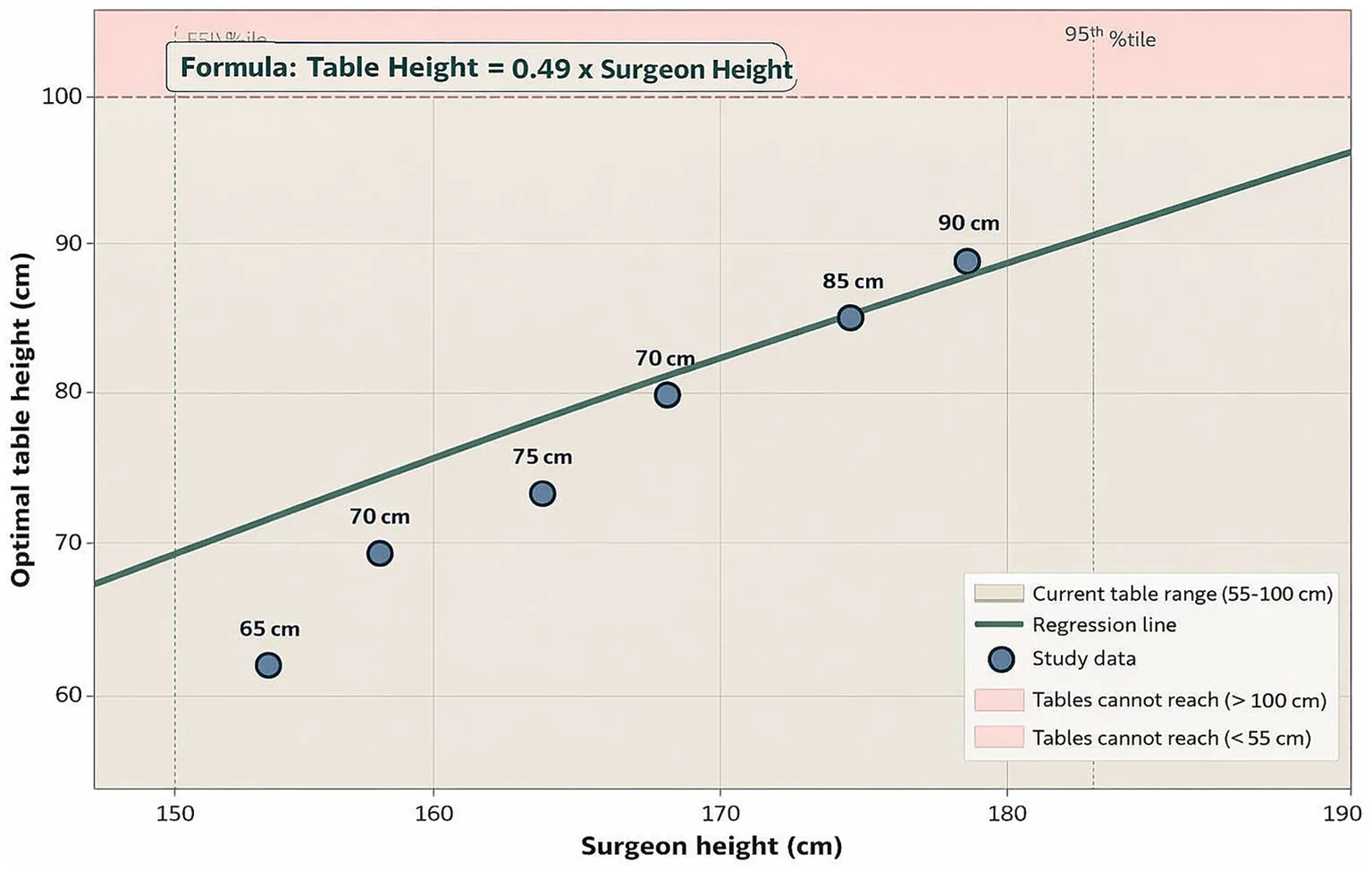
Relationship between surgeon height and optimal table height.

Surgical table height plays a pivotal role in surgeon ergonomics, particularly in prolonged spine procedures where awkward postures contribute to musculoskeletal disorders (MSDs). Table 5 lists the six individual studies underlying the surgery-type ranges already summarized in Table 3 and Section 3.5, with a focus on spine-related outcomes; consistent with those findings, current tables (range: 55–100 cm) fail to achieve the required ranges spanning 29–122 cm. This mismatch heightens risks of lumbar flexion and cervical hyperextension, common in naked-eye spine surgery. As another approach, the linear formula (Figure 9): Table Height = 0.49 × Surgeon Height (R^2^ high), validated across anthropometrics can reduce MSD incidence by 30–50% in analogous procedures (32).

**Table 5:** Summary of optimal surgical table heights by study and type of surgery.

Surgery type	*n*	Optimal height (cm)	Key metric	Source
Laparoscopy	21	0 to −10 cm elbow	Elbow height 64–77	(17)
Laparoscopy	8	0.7–0.8 × elbow	Elbow 29–69	(19)
Laparoscopy	25	0.49 × surgeon ht.	Table ht. 80–86	(32)
HALS	10	Elbow −5 cm	Elbow 57–99	(21)
Laparoscopy	2	Thigh level	Abdomen 29–122	(23)
ORL seated	5	−12.5 to +2.5 cm elbow	Elbow 64–77	(14)

This table lists the per-study sample sizes and key metrics underlying the consolidated surgery-type ranges presented in Table 3.

## 4 Discussion

Our review supports the current view that surgeon ergonomics should be understood as a systems problem rather than a series of isolated posture errors. Recent systematic reviews across orthopaedics, spine surgery, hepatopancreatobiliary surgery, ophthalmology, and plastic surgery consistently show a high burden of work-related musculoskeletal symptoms, most often affecting the neck, lower back, shoulders, and upper extremities (1, 40, 41). This broader literature also agrees with our findings that discomfort is not only common but consequential, because it is associated with treatment seeking, concern about disability, time away from work, caseload reduction, and early thoughts about career modification (9, 41, 42).

The cervical spine findings in our review are particularly important because recent evidence places neck loading at the center of surgeon injury risk. A 2024 systematic review of cervical dysfunction in surgeons found that repetitive motion, prolonged static neck posture, and non-neutral visual targets are major drivers of cervical strain, with reported symptom prevalence ranging widely but remaining consistently substantial across specialties (43). This aligns closely with our observation that low neck flexion and minimal rotation represent the safest working zone. Mechanistically, once the head moves anterior to the trunk, the cervical extensors and upper trapezius must generate sustained isometric force to counter the increasing flexion moment, which raises muscle activation, reduces local recovery time, and accelerates fatigue during long cases (40, 43). Our findings therefore fit the contemporary literature in suggesting that the neutral zone is not simply a descriptive ideal, but a biomechanical threshold beyond which muscular demand rises quickly, and cumulative tissue loading becomes more likely (42).

The upper limb findings in our synthesis are also consistent with current evidence. Recent reviews of open, laparoscopic, and robotic surgery note that shoulder abduction, unsupported forearms, forceful grip, and repetitive wrist deviation all contribute to pain and fatigue, especially when long instruments transmit load away from the surgeon’s natural working envelope (44). This explains why our review found benefits from reduced shoulder abduction, more favorable elbow angles, and accessories such as arm or body support. From a mechanistic standpoint, as the hands move farther from the trunk and the shoulders abduct, the deltoid and trapezius must sustain greater stabilizing activity while the forearm flexors and extensors manage grip and fine motor control, creating a combined static and repetitive load pattern that is well suited to fatigue and overuse (45, 46).

Our table height findings are also strongly supported by recent literature. Contemporary reviews continue to recommend aligning the operative target near elbow height and adapting table position to the surgeon rather than forcing the surgeon to adapt to the table (42, 44). This is important because table height has downstream effects on the entire kinetic chain. If the table is too high, the surgeon compensates through shoulder elevation and wrist extension, whereas if it is too low, cervical flexion and trunk inclination increase to preserve visual access and manual precision (1, 40). Our conclusion that currently available adjustment ranges are often insufficient is therefore in line with recent recommendations calling for finer and broader intraoperative adjustability (42).

The literature on visualization methods also maps well onto our results. Recent comparisons of robotic and conventional laparoscopy show that robotic systems often improve surgeon posture and reduce localized muscle fatigue, although some studies also report higher mental demand or stress and note that not everybody region benefits equally (46–48). A 2025 assessment of four robotic platforms found that knee, elbow, and back posture were generally within ergonomic recommendations, but hip and neck alignment remained suboptimal in several systems, which mirrors our finding that better technology does not eliminate the need for ergonomic optimization (49). Similarly, recent exoscope literature in spine surgery reports improved perceived ergonomics and visual freedom compared with the operating microscope, largely because the visual target is relocated to a more favorable line of sight, although concerns remain regarding depth perception, workflow adaptation, and long-term effectiveness (50). In mechanistic terms, these technologies help when they decouple vision from forced head posture and allow the surgeon to keep the torso, neck, and upper limbs closer to their natural alignment (51).

One of the most clinically relevant themes in the recent literature is that ergonomic risk is not evenly distributed across surgeons. A 2023 systematic review and meta-analysis found that hand size, stature, and sex all affect tool usability, discomfort, and task performance in traditional laparoscopy (52). A subsequent systematic review focused on female surgeons and surgeons with small hands reported frequent difficulty, pain, and injury with hand held instruments (53), while a separate review of unique surgeon populations concluded that small handed surgeons, pregnant surgeons, and trainees face distinct ergonomic barriers that are not well addressed by current equipment design (54). Newer original studies reinforce this point by showing that surgeons with smaller glove sizes report greater difficulty with common instruments and by demonstrating higher forearm muscle activation during laparoscopic instrument use in smaller handed surgeons (55–57). Our findings on anthropometry therefore fit a growing body of evidence that the operating room has historically been configured around a narrow physical template, which can systematically disadvantage shorter surgeons and those with smaller hands (58).

The interventional literature is still developing, but it provides useful direction. The UPRISE pilot study showed that wearable posture feedback can reduce time spent in high-risk posture in the pediatric operating room (59), and a Canadian pilot study in ophthalmology residents found stable or improved pain scores alongside posture improvement after wearable feedback and education (60). A recent systematic review and meta-analysis of physical activity interventions in surgeons found that active microbreaks, passive microbreaks, and exercise based strategies can reduce musculoskeletal pain, although the certainty of evidence remains low (61). Educational studies also suggest that ergonomics training is feasible in residency, but formal curricula remain uncommon despite high symptom prevalence among trainees and practicing surgeons (9, 58, 62). These findings suggest that prevention should move beyond passive awareness and toward measurable interventions that combine operating room redesign, wearable monitoring, structured training, and brief intraoperative recovery strategies.

This review deliberately prioritized objective biomechanical and electromyographic outcomes over self-reported measures. Nevertheless, subjective and survey-based data retain a complementary role: national and institutional surveys, such as those describing surgical ergonomics training and awareness in Italy (9) and interprofessional assessment of musculoskeletal pain among surgery residents (62), continue to capture real-world exposure, symptom burden, and adherence to ergonomic recommendations that laboratory- and simulation-based measures may under-represent. Future syntheses would benefit from explicitly integrating both data types rather than treating them as competing evidence streams.

### 4.1 Limitations

The main limitation of our review is the heterogeneity of the underlying literature. The highlight limitation is variations in study design, specialty, simulation versus live operating room setting, ergonomic instruments, and outcome definitions, which limits direct comparison and makes universal threshold setting difficult. Many studies also rely on small samples, short tasks, self-reported symptoms, or older technology, so some findings may not fully translate to modern integrated suites, newer robotic systems, or updated visualization platforms. In addition, objective biomechanical measures are not always linked to long term clinical outcomes, so the degree of angular or electromyographic improvement required to prevent future injury remains incompletely defined. A further limitation concerns the potential for sponsor-related bias: two authors (JW, EO) are employees of Mizuho OSI, a manufacturer of surgical tables, and GM received compensation from this company for statistical analysis and medical writing. Because the central practical conclusion of this review, that current surgical tables cannot meet the evidence-based height requirements, aligns with this sponsor’s product category, readers should weigh this conclusion, and particularly the 29–122 cm figure derived from a single small simulation study, with appropriate caution. Finally, our search was restricted to peer-reviewed literature indexed in PubMed, Scopus, and Web of Science and did not include a systematic search of grey literature (e.g., conference proceedings, theses, or preprints), which may have introduced publication bias and omitted relevant unpublished data.

### 4.2 Future directions and implications

Future research should prioritize multicenter live operating room studies that combine motion capture, electromyography, workload assessment, and follow up symptom data within the same protocol. Comparative trials testing modifiable interventions such as broader table height ranges, individualized instrument sizing, integrated arm support, wearable biofeedback, and structured microbreak programs rather than relying mainly on descriptive surveys are needed. From a practical standpoint, our findings support procurement and training policies that treat surgeon ergonomics as a workforce and patient safety issue. We recommend neutral positioning, adjustable operating room setup, instrument redesign for diverse users, and formal education as core components of prevention rather than optional add ons.

## 5 Conclusion

In conclusion, this review points out that safer surgical ergonomics depends on maintaining neutral cervical and trunk posture, minimizing unsupported upper limb loading, matching table height and visualization to surgeon anthropometry, and designing instruments and workspaces for a diverse surgical workforce. The field is moving from descriptive symptom surveys toward objective measurement and targeted intervention, but stronger prospective evidence is still needed before ergonomic best practice can be standardized across specialties and platforms.

## Data Availability

The original contributions presented in the study are included in the article/Supplementary material, further inquiries can be directed to the corresponding author/s.
