# Supplementary material for: Evidence-based ergonomic parameters for safer surgical practice: a systematic review
Source: Front Public Health. 2026 Aug 13;14:1872145. doi: 10.3389/fpubh.2026.1872145 (PMC13520068; doi:10.3389/fpubh.2026.1872145)
Supplement: Supplementary file 1 [file Supplementary_file_1.docx]

Supplementary table 1: databases search results.

| **Database** | **Keywords** | **Filters** |
| --- | --- | --- |
| **PubMed** | **(cervical spine OR cervical vertebra OR cervical fracture OR cervical trauma OR cervical dislocation OR vertebral fracture OR spinal fracture OR thoracic fracture OR lumbar fracture OR cervicothoracic OR thoracolumbar fracture) AND (circumferential OR 360 degree OR combined anterior posterior OR multi-approach OR dual approach) AND (repositioning OR patient flip OR position change OR table rotation OR Jackson table OR Mizuho OR single approach OR anterior only OR posterior only)** | **None** |
| **Cochrane Library** | **(cervical spine OR cervical vertebra OR cervical fracture OR cervical trauma OR cervical dislocation OR vertebral fracture OR spinal fracture OR thoracic fracture OR lumbar fracture OR cervicothoracic OR thoracolumbar fracture) AND (circumferential OR 360 degree OR combined anterior posterior OR multi-approach OR dual approach) AND (repositioning OR patient flip OR position change OR table rotation OR Jackson table OR Mizuho OR single approach OR anterior only OR posterior only)** | **Title, Abstract, Keywords** |
| **Scopus** | **( "cervical spine" OR "cervical vertebra" OR "cervical fracture" OR "cervical trauma" OR "cervical dislocation" OR "vertebral fracture" OR "spinal fracture" OR "thoracic fracture" OR "lumbar fracture" OR cervicothoracic OR "thoracolumbar fracture" ) AND ( circumferential OR "360 degree" OR "combined anterior posterior" OR "multi-approach" OR "dual approach" ) AND ( repositioning OR "patient flip" OR "position change" OR "table rotation" OR "Jackson table" OR Mizuho OR "single approach" OR "anterior only" OR "posterior only" )** | **None** |
| **WOS** | **(cervical spine OR cervical vertebra OR cervical fracture OR cervical trauma OR cervical dislocation OR vertebral fracture OR spinal fracture OR thoracic fracture OR lumbar fracture OR cervicothoracic OR thoracolumbar fracture) AND (circumferential OR 360 degree OR combined anterior posterior OR multi-approach OR dual approach) AND (repositioning OR patient flip OR position change OR table rotation OR Jackson table OR Mizuho OR single approach OR anterior only OR posterior only)** | **None** |

Supplementary Table 2a: Risk of bias assessment for the observational studies.

Supplementary Table 2b: Risk of bias assessment for the RCTs.
